# The Role of Dietary Protein and Fat in Glycaemic Control in Type 1 Diabetes: Implications for Intensive Diabetes Management

**DOI:** 10.1007/s11892-015-0630-5

**Published:** 2015-07-23

**Authors:** Megan Paterson, Kirstine J. Bell, Susan M. O’Connell, Carmel E. Smart, Amir Shafat, Bruce King

**Affiliations:** Department of Paediatric Diabetes and Endocrinology, John Hunter Children’s Hospital, Newcastle, NSW Australia; Hunter Medical Research Institute, School of Medicine and Public Health, University of Newcastle, Rankin Park, NSW Australia; Department of Paediatrics and Child Health, Cork University Hospital, Cork, Ireland; Physiology, School of Medicine, National University of Ireland, Galway, Galway, Ireland

**Keywords:** Type 1 diabetes, Fat, Protein, Carbohydrate, Glycaemia, Insulin

## Abstract

A primary focus of the management of type 1 diabetes has been on matching prandial insulin therapy with carbohydrate amount consumed. However, even with the introduction of more flexible intensive insulin regimes, people with type 1 diabetes still struggle to achieve optimal glycaemic control. More recently, dietary fat and protein have been recognised as having a significant impact on postprandial blood glucose levels. Fat and protein independently increase the postprandial glucose excursions and together their effect is additive. This article reviews how the fat and protein in a meal impact the postprandial glycaemic response and discusses practical approaches to managing this in clinical practice. These insights have significant implications for patient education, mealtime insulin dose calculations and dosing strategies.

## Introduction

Intensive insulin management and good glycaemic control are essential for the prevention of acute and long-term diabetes complications and the overall wellbeing in people with type 1 diabetes [1, 2]. Multiple daily injection (MDI) therapy involves injecting long-acting insulin once or twice daily (basal) and injections of rapid-acting insulin before each meal. This remains the most common form of insulin therapy for type 1 diabetes; however, insulin pump therapy (IPT) is becoming an increasingly popular form of therapy. An extension of MDI and IPT is flexible intensive insulin therapy, which involves calculating the mealtime insulin based on carbohydrate (CHO) content of the meal and the pre-prandial blood glucose level (BGL) [1, 3, 4]. Adjusting insulin doses based on CHO intake in intensive insulin regimes has been shown to improve glycaemic control, decrease hypoglycaemia and improve quality of life [1, 3–8]. However, even with the introduction of intensive insulin regimes, analogue insulins and CHO quantification, day-to-day glucose variability remains a major issue [9] and diabetes centres have not seen the anticipated improvements in glycaemic control [10–12]}.

One of the key factors affecting the mean BGL and HbA1c is the postprandial BGL. Postprandial BGLs have been shown to increase the risk factors for cardiovascular disease in people with type 1 diabetes [13, 14]. Hence, factors that increase the postprandial BGL have the potential to increase HbA1c and the risk of complications.

**Fig. 1:**
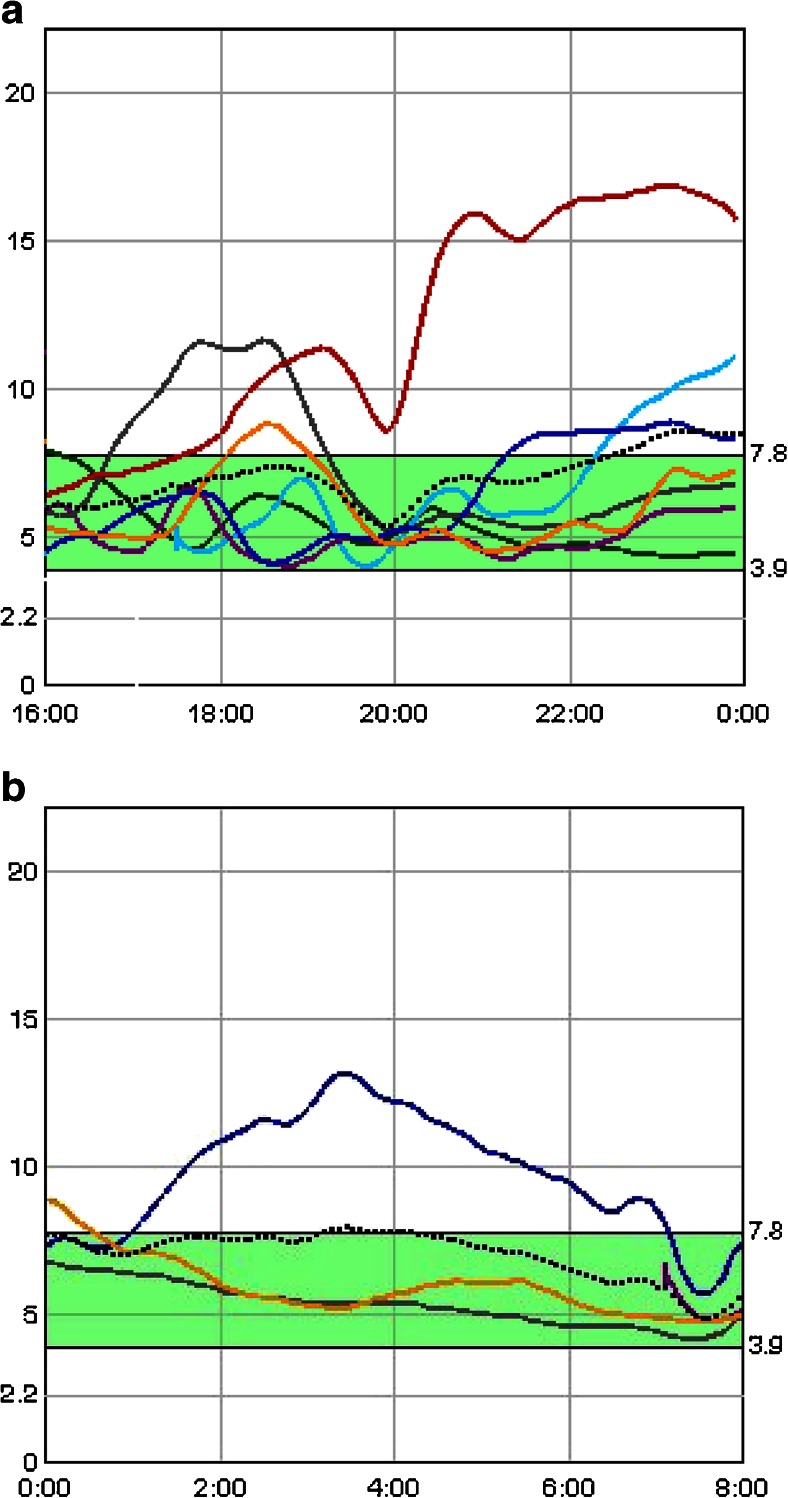
Continuous glucose sensor results demonstrating the impact of macronutrients on postprandial glycaemia. Graph A is a 10-year-old female with type 1 diabetes on insulin pump therapy who was well controlled but ate pizza at 18:00 (*red line*). The standard bolus did cover the first 2 h but then she developed extended hyperglycaemia. Graph B is an 18-year-old male with type 1 diabetes on insulin pump therapy who ate fried chicken at 22:00 (*blue line*). The standard bolus controlled the initial postprandial period but he developed hyperglycaemia 3 h later which lasted 6 h. (With permission from: Smart, C. Diabetes Care for Children and Young People. Diabetes and Primary Care 2013, 2(2):71–73) [15]

Although CHO is the predominant macronutrient affecting postprandial BGL, recent research has shown that dietary fat and protein can also significantly impact the postprandial glycaemic profile (Fig. 1) [16, 17, 18••, 19••], and thus adjusting the prandial insulin dose for these macronutrients may be beneficial [16, 20••].

Dietary CHO is digested into glucose (and other monosaccharides) and transported into the bloodstream, where it directly raises the blood glucose concentration. Since other macronutrients, including protein and fat, have little direct impact on postprandial glycaemia in healthy subjects, they were essentially ignored for determining prandial insulin doses. Early research in the 1980s confirmed this relationship between CHO and insulin therapy in type 1 diabetes using an artificial pancreas [21–23]. Around the same time, there was evidence to suggest that high-fat and high-protein meals influence exogenous insulin requirements in type 1 diabetes, however these were largely overlooked in the enthusiasm for carbohydrate counting ([24, 25].

Optimising insulin dosing for fat and protein is an important clinical issue given fat and protein are recommended to comprise a combined 45–50 % of daily energy intake (∼30 % for fat and 15–20 % for protein) [26, 27]. Furthermore, low-CHO diets (with consequently higher intakes of fat and protein) have recently received a great deal of media attention and are becoming increasingly popular.

This article will therefore review the evidence regarding the glycaemic impact of fat and protein and discuss the clinical implications and mealtime insulin dosing strategies in the management of type 1 diabetes.

## Fat

### Glycaemic Impact

Unlike proteins or CHO, fat is rarely consumed in isolation as a single macronutrient, therefore the impact of fat is usually considered in terms of its ability to modify the CHO-induced blood glucose response.

In healthy people and people with type 1 diabetes, adding fat to a CHO meal reduces the glycaemic response in the early postprandial period (first 1–3 h) but extends the glycaemic response over hours. Using continuous glucose monitoring (CGM) profiles in 24 healthy non-diabetic adults, Freckmann et al. demonstrated that meals with a higher fat content induced a smaller increase and a slower decrease of postprandial glucose concentrations than a high-carbohydrate low-fat meal [28].

As with healthy individuals, the addition of fat tends to decrease the glycaemic response in the early postprandial period in people with type 1 diabetes, and thus increases the risk of hypoglycaemia. However, in the late postprandial period, high-fat meals often cause significant hyperglycaemia lasting for several hours following the meal. This hyperglycaemia is problematic for good glycaemic control, particularly since high-fat meals are often consumed for the evening meal when prolonged hyperglycaemia may not be detected until the following morning. A recent study of the dietary factors associated with nocturnal hypoglycaemia in adults has shown a significant association with post-dinner fat intake and higher rates of nocturnal hyperglycaemia occurrence [29].

Several studies looking at the effects of dietary fat have been published in recent years. We have shown that the addition of 35 g of dietary fat to 30 g of CHO initially reduces the glycaemic excursion for up to 90 min following the meal but then significantly increases the postprandial glycaemic response from 3 h onwards, with the BGL increased by 2.3 mmol/L at 5 h [18••]. In an insulin clamp study, Wolpert et al. also demonstrated that a high-fat meal (50 g of fat) causes significant hyperglycaemia over 5 h, even with additional insulin infused [19••].

### Mechanisms of Action

Unlike proteins or CHO, fat is rarely consumed on its own as a single macronutrient. Triacylglycerols (TAGs) constitute more than 90 % of lipids in the human diet, and this review will focus on their role in the management of BGLs.

TAGs can influence the glycaemic response in type 1 diabetes by (1) gluconeogenesis of glycerol, (2) a direct effect of free fatty acids (FFA), (3) effects on other hormones and (4) effects on gastric emptying.

1. *Gluconeogenesis from fat*
  Dietary fat is not converted to glucose in any substantial amounts. Fatty acids are metabolised to ketones which cannot be used in gluconeogenesis [30]. In contrast, glycerol from the hydrolysis of TAGs can be metabolised to pyruvate and synthesised into glucose [30]. However, glycerol only makes up 5–15 % of the weight of TAGs (depending on which fatty acids are in the TAG) and only part of the glycerol would be converted to glucose with the rest entering the glycolytic pathway [31].
2. *Direct effect of fatty acids*
  Circulating FFAs have a direct effect on the beta cells causing increased glucose-stimulated insulin secretion, which has been attributed to interactions with a G protein-coupled receptor [31]. Additionally, FFAs are the ligand for peroxisome proliferator-activated receptors (PPARs). The thiazolidinediones are PPARγ agonists which are used in the management of type 2 diabetes. See Grygiel-Gorniak for a review of PPARs [32].
3. *Effects of fats on other hormones*
  Dietary fat has been shown to alter the release of other hormones which impact on glycaemic regulation including glucagon, glucagon-like protein 1 (GLP-1), gastric inhibitor polypeptide (GIP) and ghrelin.
  In healthy subjects, when fat is consumed alone, postprandial glucagon and GLP-1 levels increase, but if the fat is consumed with CHO, the glucagon and GLP-1 levels do not increase [33]. Ladbroke et al. found that when adolescents with type 1 diabetes consumed a high-fat and high-CHO meal, GLP-1, GIP and ghrelin increased [34]. The impact of dietary fat on other hormones is beyond the scope of this article, and the reader is referred to other reviews [35].
4. *Effect of fat on gastric emptying*
  The duodenal delivery of nutrients is a major determinant of BGL. As the CHO absorption capacity of the small intestine far exceeds the duodenal delivery, gastric emptying is a rate-limiting step in glucose absorption [36, 37]. As the stomach empties at a constant energy rate (8.4 kJ/min), addition of fat to a meal will delay the rate of gastric emptying of CHO [38]. Addition of fat to a CHO meal delayed and reduced the glycaemic and insulinemic responses in healthy individuals [39]. Clegg et al. showed that in healthy subjects, the chain length and saturation of fat is important and changes the glycaemic response to a meal [40].

The impact of dietary fat on gastric emptying of the subsequent meal regardless of its content also needs consideration, as dietary fat from breakfast has been shown to delay the rate of gastric emptying of the subsequent lunch meal [41]. Furthermore, intake of high-fat diet for 1 week desensitises gastrointestinal transit and leads to a more rapid gastric emptying [42].

Because of fats impact on gastric emptying, modification of dietary fat content is used in the management of diabetes-related gastroparesis.

## Protein

### Glycaemic Impact

In individuals without diabetes, dietary protein does not alter postprandial glycaemia [43], however, it stimulates a significant postprandial insulin response, which is required for amino acid uptake [44]. In order to maintain euglycaemia, protein concurrently stimulates glucagon secretion, thereby promoting hepatic glucose release and regulation of BGL [45–47].

In contrast, when people with type 1 diabetes consume protein, there is an increase in postprandial BGLs and insulin requirements. Studies comparing the postprandial glycaemic responses to standard meals with meals containing between 28 and 57 g of additional protein have demonstrated significantly higher postprandial glycaemic excursions and insulin requirements in the 2–5 h postprandial period in type 1 diabetes [17, 18••, 45, 48]. More recently, our group looked at the glycaemic impact of ingested protein only (independent of CHO and fat) in children and young adults with type 1 diabetes [49]. Consumption of ≥75 g of protein resulted in a significantly delayed, sustained, elevated, postprandial glycaemic excursion from 180 to 300 min compared with a control drink (water). The peak BGL from ≥75 g of protein was similar to that of 20 g of glucose given without insulin; however, the shape of the glycaemic response was significantly different. Following the ingestion of protein, the BGL only began to rise after 100 min and reached the peak BGL after 5 h.

These findings demonstrate that for people with type 1 diabetes the addition of ≥28 g of protein to a mixed meal or consuming ≥75 g of protein alone is likely to result in significant and sustained postprandial hyperglycaemia commencing in the late postprandial period (2–3 h) and continuing beyond 5 h. Clinically, this is important when determining whether additional insulin is required and, importantly, how to safely calculate and distribute these increased doses.

The sustained postprandial hyperglycaemic effect of protein could potentially be utilised to decrease nocturnal hypoglycaemia, however, research has shown that high-fat meals (which also have sustained postprandial hyperglycaemia) did not prevent nocturnal hypoglycaemia [50]. Hence, further research is required to determine if high-protein meals would decrease nocturnal hypoglycaemia.

### Mechanisms of Action

Two mechanisms have been proposed by which dietary protein may cause delayed and sustained postprandial glycaemic excursions in people with type 1 diabetes: (1) the alteration of hormones which affects glucose homeostasis and (2) the conversion of amino acids to glucose by gluconeogenic pathways.

1. Alteration of hormones which affect glucose homeostasis
  Ingestion of a high-protein meal has been shown to increase circulating plasma glucagon in both healthy people and people with type 1 diabetes [45–47]. In people without diabetes, the concurrent stimulation of glucagon counters the effects of protein-induced insulin release (whilst still allowing insulin to transport amino acids into the cells), and thus, there is minimal impact on postprandial glycaemia. However, in people with type 1 diabetes, this increase in plasma glucagon in the absence of sufficient insulin has been shown to cause postprandial hyperglycaemia [45, 51].
  Meal protein content also influences a number of other hormones, such as cortisol, growth hormone, IGF-1 and ghrelin, but how these hormonal changes subsequently influence postprandial glucose levels is poorly understood. High-protein meals produce increased cortisol concentrations [52], which in turn may increase insulin resistance and insulin requirements. Meals that are high protein and low CHO produce increased growth hormone levels; however, when the meal contains significant amounts of CHO, growth hormone is unchanged or decreased [53]. Meals high in protein increase circulating IGF-1 levels and decrease ghrelin levels [54].
2. Conversion of amino acids to glucose (gluconeogenesis)
  Amino acids can act as an energy source by being converted to glucose (glucogenic amino acids) or into ketone bodies (ketogenic amino acids). There are 20 amino acids that are encoded by the nuclear genes of eukaryotes, 13 are exclusively glucogenic, 2 are exclusively ketogenic and 5 are glucogenic and ketogenic. Hence, 18 out of the 20 amino acids (90 %) can be converted to glucose. The exclusively glucogenic amino acids are glycine, serine, valine, histidine, arginine, cysteine, proline, alanine, glutamate, glutamine, aspartate, asparagine and methionine. The amino acids that are both glucogenic and ketogenic are isoleucine, threonine, phenylalanine, tyrosine and tryptophan. The exclusively ketogenic amino acids are leucine and lysine [55]. When circulating insulin levels are inadequate, there is an uptake of glucogenic amino acids by the liver, thereby increasing gluconeogenesis and resulting in increased plasma BGL [56–58].

A study by Khan et al. showed that 50 g of ingested protein resulted in an additional 9.7 +/− 5.7 g of glucose in the circulation over 8 h in healthy adult males [43]. This demonstrates that by weight, 19.4 % of the total amount of protein ingested can be converted to glucose. However, considering only the amount of glucose that enters the circulation ignores the protein-induced hormonal response (as discussed above) and that some additional insulin may be needed to counter the resulting decrease in insulin sensitivity.

Alternatively, Pańkowska et al. have proposed that 100 kcal of fat/protein requires the equivalent amount of insulin as 10 g of glucose [16, 59]. Using this ratio (protein contains 4.1 kcal/g), 100 kcal of protein = 24.4 g of protein = 10 g of glucose. This is approximately twice the amount of glucose that Khan and colleagues found was produced from protein in healthy volunteers [43]. This apparent discrepancy may be explained because Khan et al. measured the amount of glucose produced, whereas Pańkowska et al. used clinical response (which would account for glucose production and hormonal responses). Kordonouri et al. used the Pańkowska equation in children with type 1 diabetes using IPT and found that although glycaemic control was improved, the risk of postprandial hypoglycaemia was increased threefold [20••]. This suggests that further research is required to determine how much additional insulin is required to cover the protein component of a meal.

## Combining Fat and Protein

Although both protein and fat can independently increase postprandial glycaemia in type 1 diabetes, most meals contain both fat and protein so it is important to consider the impact of these nutrients in combination. Our research has revealed that when both fat and protein are added to a meal, their glycaemic impacts are *additive*. The addition of 30 g of fat to 30 g of CHO increased glycaemia by 1.8 mmol/L at 5 h, and similarly, the addition of 40 g of protein to the same amount of CHO increased glycaemia by 2.4 mmol/L at 5 h [18••]. However when both nutrients were added to the meal, the postprandial glycaemic response was increased by 5.4 mmol/L, the sum of the effects of the two nutrients individually [18••].

Our results are supported by other studies in the literature. Neu et al. showed that the addition of 82 g of protein and 33 g of fat to 70 g of protein increased the glucose response by 40 % over 12 h, with significant differences in glycaemia seen from 4 to 12 h and the peak difference observed 6 h following the meal [60]. Similarly, Garcia-Lopez et al. found that the glucose profile varied significantly with time when both fat and protein were added to a CHO meal. When the low protein/fat meal was consumed, the peak BGL was reached after 1 h and returned to baseline by 3 h. However, when fat and protein were added to the same meal, the peak BGL was delayed by 30 min but stayed elevated for the remainder of the 3 h test session and did not return to baseline [48].

## Dosing Insulin for Fat and Protein

As both fat and protein increase postprandial glycaemia beyond that of CHO alone, effective strategies to adjust insulin doses for these macronutrients are required. It is important to note that in practice, people consume mixed meals containing combinations of CHO, protein and fat, and therefore, the overall impact of these macronutrients on postprandial BGLs needs to be considered rather than the effect on individual nutrients. When determining how to administer insulin for meals with varying CHO, fat and/or protein contents, it is important to consider the effects in terms of managing both the *early* and *late* postprandial glycaemic responses.

### Controlling the Immediate Postprandial Glycaemic Response

Managing the early postprandial period is the first step in adjusting mealtime insulin doses, as changes implemented here will have flow-on effects for the late postprandial period.

For high CHO meals, controlling the immediate postprandial glycaemic rise is important as an increased initial glucose spike can increase the entire postprandial excursion [61]. For example, an increase in the early postprandial response by 2 mmol/L, with a consequential increase in BG at all time points, could result in a 2–3× fold greater glycaemic excursion. This is especially true for meals also containing significant amounts of fat and protein, which will further increase the late postprandial period.

Changing the timing of the insulin bolus to minimise the immediate postprandial response has been investigated, however, not in the context of high-fat and/or high-protein meals. Although the immediate postprandial glucose rise is blunted after a high-fat meal, there is still a need for insulin to cover the CHO component of the meal [18••]. Therefore, in the author’s opinion, delaying the insulin injection for high-fat meals would cause unacceptable postprandial hyperglycaemia. Research looking at the timing of the insulin bolus has shown that giving the insulin bolus before starting to eat gives superior glycaemic control compared to delaying the insulin bolus [61–63]. Conversely, for high-CHO meals also containing fat and protein, two studies have shown that dosing insulin 15 min prior to the meal was able to improve control over the initial postprandial spike, significantly reducing the peak BGL [62, 64]. However, in practice, dosing 15 min prior to a meal may prove too onerous for some patients and may result in insulin doses being forgotten. As with all diabetes management recommendations, advice should be tailored to the individual.

For patients using IPT, the insulin delivery pattern can be adjusted to better match insulin requirements. The bolus for a meal can be given as a standard bolus (where the entire dose is given within 5 min), an extended bolus (where the dose is infused at a constant rate over a chosen number of hours) or a combination bolus (where a proportion of the dose is delivered immediately and the remainder is infused continuously as an extended bolus). However, not all boluses are able to control the immediate postprandial rise [65, 66]. Chase et al. found that extended boluses did not control the immediate postprandial glucose rise with a high-fat meal [65]. Lopez et al. attempted to optimise the amplitude (height of the wave) of the extended bolus but was not able to control the immediate postprandial glucose rise compared to the standard bolus [66].

Hypoglycaemia in the early postprandial period is also a concern for low-CHO meals containing significant amounts of protein and/or fat, with patients often reporting hypoglycaemia soon after the meal when insulin is dosed upfront. This is likely due to mismatch between the rapid-acting insulin and the delayed gastric emptying following fat and the delayed glycaemic responses for both protein and fat. For these meals, the initial insulin dose needs to be reduced upfront but potentially increased in the latter phase, with the total dose delivered over an extended period to match the food absorption.

Changing the type of insulin delivered is an option for reducing the risk of early hypoglycaemia with MDI. To our knowledge, there are no studies comparing regular insulin with rapid-acting insulin (such as aspart or lispro insulin) for high-fat and/or high-protein meals. However, in theory, regular insulin with its slower onset of action and longer duration of action may offer an advantage in meals high in fat where gastric emptying is delayed and the initial postprandial glucose rise is blunted [18••].

### Controlling the Late Postprandial Glycaemic Response

Fat and protein cause increased glycaemia continuing to 6–12 h after meal ingestion [16, 18••, 20••, 50]. To cover this extended glycaemic effect of fat and/or protein, the duration of the insulin effect needs to be extended and total dose may need to be increased.

There is much debate over how additional insulin doses should be calculated and how much protein or fat needs to be consumed before insulin doses need to be adjusted. Two different algorithms have been proposed to calculate insulin for meal fat and protein content. Pankowska et al. have proposed that the insulin dose for 10 g of CHO should be equivalent to the insulin needed for every 100 kcal of fat/protein. Alternatively, Brand-Miller et al. have proposed a food insulin index, which is a measure of the postprandial insulin response to foods in healthy subjects, as a basis for determining mealtime insulin doses in type 1 diabetes [12, 67, 68]. However, both of these methods have resulted in significant postprandial hypoglycaemia and require further research.

Closed-loop studies have suggested that for high-fat and/or high-protein meals, the insulin dose needs to be increased by 42 % to as much as 125 %. Wolpert et al. revealed a mean insulin dose increase of 42 % was needed for a high-fat meal (60 g fat) compared to the low-fat meal (10 g fat), however, given patients still experienced significantly hyperglycaemia following the high-fat meal, this dose is likely to be an underestimation [19••]. Importantly, there was a wide variation in the dose adjustment needed to cover the high-fat meal (range −17 to +108 %), highlighting the importance of individualised advice. A subsequent study by this group used predictive modelling to optimise the insulin dose in patients using insulin pump therapy for the addition of 35 g of fat and 32 g of protein to a meal, finding a mean insulin dose increase of 78 %, using a combination-wave with 30/70 % split over 2.4 h [69]. Similarly, there was a wide variation in the total dose increase (+38 to +125 %) and the optimal split and duration (range 20/80 % to 50/50 % and 2–3 h).

Since rapid-acting insulin given as a standard bolus will not last long enough to cover the long tail caused by protein and fat, altering the insulin delivery pattern (i.e., bolus type) may assist with controlling late postprandial hyperglycaemia in patients using IPT. Extended boluses are also ineffective for high-protein/fat meals, as they are unable to control the immediate postprandial BGL rise [65, 66, 70], even though the rate of the rise is blunted [18••]. Currently, the most effective form of insulin pump bolus for meals containing protein and/or fat is the combination-wave bolus [65, 70, 71]. Jones et al. demonstrated that compared with a standard bolus, a combination-wave bolus significantly reduced the mean BGL and improved the amount of time spent within the normal BGL range for a high-fat pizza meal [71]. Similarly, Chase et al. also found that the combination-wave gave the best postprandial glycaemic control following a high-fat pizza and tiramisu meal [65]. To date, there are no studies looking at the optimal split of insulin between the immediate and extended bolus components for combination-wave boluses. However, studies have used 50 % of insulin given immediately and 50 % given as the extended bolus, and this can be used as an appropriate starting point in practice and adjusted as needed. The duration has been found to have an improved glycaemic profile when the duration of the bolus is increased from between 4 and 8 h [5].

Extending the insulin action time is more problematic for patients using MDI. Theoretically, the insulin injection could be given as a split bolus with two separate injections: one injection prior to the meal and the other given 1 h following the meal. However, this method needs to be investigated to determine its efficacy in practice.

For small meals and snacks, large adjustments of insulin doses may not be required. Wilson et al. compared a low-fat (30 g CHO, 2.5 g protein, 1.3 g fat; 138 kcal) and a high-fat (30 g CHO, 2 g protein, 20 g fat; 320 kcal) snack at bedtime and showed that during an 8-h overnight period, the average BGL following the low-fat snack was 8.7 and 9.5 mmol/L following the high-fat snack [50]. In this scenario with a high-fat snack (rather than a large meal), a standard bolus for the CHO component and a temporary 10 % increase in basal overnight would appear appropriate.

## Conclusion

Although CHO is the principal factor influencing glycaemic response to a meal, dietary fat and protein have a major impact on postprandial glycaemia in people with type 1 diabetes, from 2 to 8 h after ingestion. Insulin strategies to control the glycaemic impact of high-fat and high-protein meals require further investigation, but the literature suggests increasing the insulin dose and delivering the insulin using a combination-wave over at least 2 h improves the post prandial glycaemic control. Further research is required to produce a pragmatic approach to dietary fat and protein that can be utilised in clinical practice.
